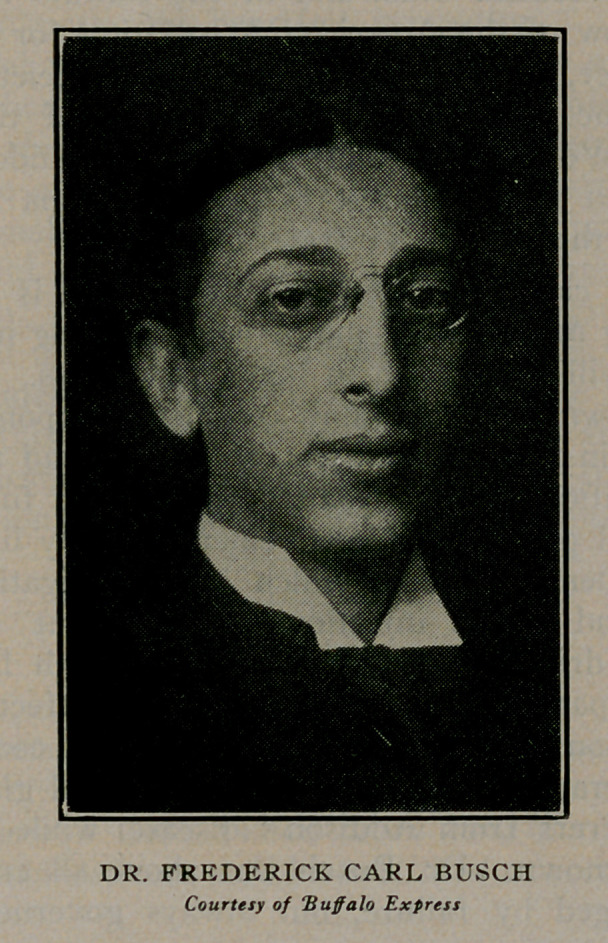# Frederick Carl Busch

**Published:** 1914-02

**Authors:** 


### Frederick Carl Busch

1873-1914—AN APPRECIATION

When the grim destroyer on January 3, 1914 swept Frederick Carl Busch into eternity, the profession lost a master-mind and medicine a faithful servant. Dr. Busch died at the age of forty, from a malignant growth in the bladder, for the treatment of which he went to Baltimore early in November, 1913, where he was unsuccessfully exposed to a massive dose of radium.

Frederick Carl Busch was born in Buffalo December 12, 1873, of a prominent Erie County German-American family, his father being Frederick Busch, now deceased, and his mother Katherine Layer; he leaves one brother, George, and one sister, Mrs. James Trounce, both of Buffalo. On June 22, 1898 he married Edith M. Fletcher of Middleport, New York, who with their son, Addison Fletcher, aged seven, survives him.

In his early years Dr. Busch attended No. 16 school in Buffalo, graduating from the Central High in 1890. He received

DR. FREDERICK CARL BUSCH

*Courtesy of Buffalo Express*

the degree of Bachelor of Science from Cornell University in 1895, and that of Doctor of Medicine from the University of Buffalo in 1897. After serving as house physician, he became an alumnus of the Buffalo General Hospital in 1898; he studied under Professor Kronecker during the year 1900 at the University of Bern, Switzerland. Again in 1912-1913 he studied in the clinics at Hamburg, Berlin, Paris and London. In 1901 he accepted the Chair of Physiology in the Medical Department of the University of Buffalo, and became a member of the executive faculty in 1910. In 1912 Dr. Busch resigned his official connection with the medical school to accept the position of physician in the Buffalo Hospital of the New York State Institute for the Study of Malignant Diseases, in which were spent, as a patient, the last days of his life.

The diversity of Dr. Busch's interest is well exemplified by his connection with many medical, scientific, historical and social associations. He was a Fellow of the American Medical Association, a member of the medical societies of New York State and of the County of Erie, of the American Physiological Society, of the American Society for the Encouragement of Clinical Research, of the Buffalo Academy of Medicine, of the Roswell Park Medical Club, and of the Buffalo Medical Club; he was a Fellow of the American Association for the Advancement of Science, a member of the Buffalo Society of Natural Sciences, of the Buffalo Historical Society, of the University Club, of the Westminster Club and of the Guido Chorus; he also kept up an active interest in the Nu Sigma Nu, the Beta Theta Pi and the Sigma Xi fraternities.

The brilliant career of Frederick Carl Busch is a shining example to young men in medicine of what untiring industry properly directed will accomplish in but a few years. His research work was known and favorably commented upon not only at home but also in foreign countries, so that he had won an international reputation before reaching the age of forty. In time to come he will probably be best remembered by his revolutionary work on hemorrhage in which his investigations in blood transfusions and serum injections brought out the superior advantages of dried serums; in association with Dr. G. H. A. Clowes the preparation of this product was perfected and given to the profession in 1911 under the name of coagulose. His studies in the transplantation of healthy adrenal glands into the tissues of sufferers from Addison's disease, while not so startling is better known. Dr. Busch was above all an investigator never discouraged by failure, but always governed by a true scientific spirit.

It is as a teacher that the younger medical men knew Dr. Busch best; they not only felt the influence of his fluent, clear and incisive exposition of the complexities of physiology, but they received an inspiration for better work, closer observation and deeper thought. His cheerful optimism, his geniality and his freedom from egotistic dignity made him the personal friend of every student, while his habit of pressing home the point by a humerous twist accompanied by that winning smile endeared him to all.

Notwithstanding the pressure of his other work, Dr. Busch found time to revise several medical works in his chosen field. His laboratory manual of physiology, a valuable addition to the text books on the subject, is very popular and has a wide use. He wrote freely and discriminatingly for the medical journals and for presentation at medical meetings, his articles always had the merit of adding something to the sum of medical knowledge.

Among his many qualifications, Dr. Busch was an accomplished linguist, a pleasing raconteur, a jolly good fellow; he was widely popular in the best sense and deservedly so notwithstanding his modesty and his domestic tendencies. Those who knew him most intimately loved him loyally, for in him was no selfishness, no deceit, no dubious ways, but always generosity, honesty, frankness and heartfelt sympathy.

The general public knew Dr. Busch best as a singer. Thousands have been charmed by his splendid voice, one of rare sweetness. He came of a music-loving family, and had he chosen music as a career, with his superb baritone he would have been equally successful as an artist. He was prodigal with this wonderful talent giving of it freely wherever it might please and entertain; he seemed to feel that it was given to him only that he might use it for the enjoyment of others.

Small wonder is it then that one gives pause to note the passage out of this life of one so richly endowed and cultivated, of one so modest and unassuming, of one so universally loved as a companion, respected as a teacher and honored as a fellowman.

## Figures and Tables

**Figure f1:**